# eNAMPT Neutralization Preserves Lung Fluid Balance and Reduces Acute Renal Injury in Porcine Sepsis/VILI-Induced Inflammatory Lung Injury

**DOI:** 10.3389/fphys.2022.916159

**Published:** 2022-06-22

**Authors:** Saad Sammani, Tadeo Bermudez, Carrie L. Kempf, Jin H. Song, Justin C Fleming, Vivian Reyes Hernon, Matthew Hufford, Lin Tang, Hua Cai, Sara M. Camp, Viswanathan Natarajan, Jeffrey R. Jacobson, Steven M. Dudek, Diego R. Martin, Christof Karmonik, Xiaoguang Sun, Belinda Sun, Nancy G. Casanova, Christian Bime, Joe G. N. Garcia

**Affiliations:** ^1^ Department of Medicine, University of Arizona Health Sciences, Tucson, AZ, United States; ^2^ Department of Anesthesiology, University of California Los Angeles, Los Angeles, CA, United States; ^3^ Department of Medicine, University of Illinois at Chicago, Chicago, IL, United States; ^4^ Department of Radiology and the Translational Imaging Center, Houston Methodist Hospital and the Houston Methodist Research Institute, Houston, TX, United States; ^5^ Department of Pathology, University of Arizona Health Sciences, Tucson, AZ, United States

**Keywords:** ARDS, eNAMPT, mAb, B-lines, DAMP

## Abstract

**Background:** Numerous potential ARDS therapeutics, based upon preclinical successful rodent studies that utilized LPS challenge without mechanical ventilation, have failed in Phase 2/3 clinical trials. Recently, ALT-100 mAb, a novel biologic that neutralizes the TLR4 ligand and DAMP, eNAMPT (extracellular nicotinamide phosphoribosyltransferase), was shown to reduce septic shock/VILI-induced porcine lung injury when delivered 2 h after injury onset. We now examine the ALT-100 mAb efficacy on acute kidney injury (AKI) and lung fluid balance in a porcine ARDS/VILI model when delivered 6 h post injury.

**Methods/Results:** Compared to control PBS-treated pigs, exposure of ALT-100 mAb-treated pigs (0.4 mg/kg, 2 h or 6 h after injury initiation) to LPS-induced pneumonia/septic shock and VILI (12 h), demonstrated significantly diminished lung injury severity (histology, BAL PMNs, plasma cytokines), biochemical/genomic evidence of NF-kB/MAP kinase/cytokine receptor signaling, and AKI (histology, plasma lipocalin). ALT-100 mAb treatment effectively preserved lung fluid balance reflected by reduced BAL protein/tissue albumin levels, lung wet/dry tissue ratios, ultrasound-derived B lines, and chest radiograph opacities. Delayed ALT-100 mAb at 2 h was significantly more protective than 6 h delivery only for plasma eNAMPT while trending toward greater protection for remaining inflammatory indices. Delayed ALT-100 treatment also decreased lung/renal injury indices in LPS/VILI-exposed rats when delivered up to 12 h after LPS.

**Conclusions:** These studies indicate the delayed delivery of the eNAMPT-neutralizing ALT-100 mAb reduces inflammatory lung injury, preserves lung fluid balance, and reduces multi-organ dysfunction, and may potentially address the unmet need for novel therapeutics that reduce ARDS/VILI mortality.

## Introduction

The adult respiratory distress syndrome (ARDS) is a life-threatening condition caused by diverse inciting stimuli including the SARS-CoV-2 coronavirus producing a world-wide COVID-19 pandemic (Garg et al., 2020). Mechanistic concepts of ARDS pathobiology implicate the involvement of pathogen-activated, evolutionarily-conserved innate immunity inflammatory pathways and pathogen recognition receptors (PRRs) (Imai et al., 2008; Gong et al., 2020). PRRs, designed for infection containment, are also triggered by mechanical ventilation-generated mechanical stress leading to ventilator-induced lung injury (VILI) (Hong et al., 2008; Camp et al., 2015; Quijada et al., 2021). Activation of PRR inflammatory cascades profoundly increases levels of inflammatory cytokines that contribute to unremitting increases in vascular permeability, an essential ARDS pathophysiologic feature that culminates in alveolar flooding, severe hypoxemia, multi-organ edema/dysfunction (MOD) and death (Bime et al., 2021). While SARS-CoV-2 vaccines and anti-SARS-CoV-2 drugs are of obvious utility, neither strategy addresses ARDS/VILI-induced unchecked inflammation and MOD. Thus, there remains an enormous unmet need for effective FDA–approved pharmacotherapies that reduce the staggering elevated ARDS mortality rates (Garg et al., 2020).

In prior work, we showed that eNAMPT (extracellular nicotinamide phosphoribosyltransferase) is a highly druggable ARDS target (Ye et al., 2005; Hong et al., 2008; Sun et al., 2014; Camp et al., 2015; Bime et al., 2019; Quijada et al., 2021; Bermudez et al., 2022) whose plasma levels are linked to ARDS severity and mortality (Ye et al., 2005; Hong et al., 2008; Sun et al., 2014; Camp et al., 2015; Bime et al., 2019; Quijada et al., 2021; Bermudez et al., 2022). eNAMPT is encoded by *NAMPT* whose expression is induced by ARDS stimuli (hypoxia, trauma, infection, mechanical stress) (Adyshev et al., 2014; Sun et al., 2014; Elangovan et al., 2016; Chen et al., 2017; Sun et al., 2020; Quijada et al., 2021; Bermudez et al., 2022). *NAMPT* promoter SNPs drive eNAMPT plasma levels and confer increased risk of ARDS severity and death (Ye et al., 2005; Bajwa et al., 2007; Sun et al., 2014; Elangovan et al., 2016; Bime et al., 2019). As an intracellular enzyme, iNAMPT regulates NAD biosynthesis (Revollo et al., 2007), however, when secreted into the circulation, eNAMPT ligates Toll–like receptor 4 (TLR4) (Camp et al., 2015) to function as a DAMP protein (tissue damage-associated molecular pattern) and master regulator of evolutionarily-conserved NFkB-driven inflammatory cascades that are involved in ARDS/VILI pathobiology (Camp et al., 2015; Quijada et al., 2021; Bermudez et al., 2022). Importantly, a humanized eNAMPT-neutralizing mAb, ALT-100, given concomitantly with LPS challenge but prior to VILI exposure profoundly reduces inflammatory lung injury and plasma cytokine levels in LPS/VILI- exposed mice and rats (Quijada et al., 2021; Bermudez et al., 2022) indicating eNAMPT directly participates in ARDS/VILI pathobiology.

The unmet need for FDA-approved ARDS therapies is linked to the challenge of ARDS heterogeneity, and the harsh reality that successful therapeutic strategies in rodents often fail to successfully translate in human clinical trials (Uhlig and Kuebler, 2018; Ware et al., 2020). Preclinical rodent ARDS models rarely use concomitant VILI exposure and invariably use timing of therapeutic administration with injury onset, i.e., not after established lung injury. We recently utilized a clinically-relevant porcine septic shock/VILI model to show IV delivery of the eNAMPT-neutralizing ALT-100 mAb 2 h after initiation of injury, significantly reduced lung inflammatory injury and improved respiratory dynamics (Bermudez et al., 2022). The primary goal of the present study was to extend these findings and uniquely evaluate the protection offered when ALT-100 mAb is delivered 6 h post the onset of injury. A second, highly clinically-relevant goal was to assess ALT-100 mAb capacity to preserve lung fluid balance and to reduce acute kidney injury (AKI), an index of MOD. These studies address a critical gap in ARDS therapeutic drug development and confirmed eNAMPT is a highly druggable ARDS target. The eNAMPT-neutralizing ALT-100 mAb directly mitigates the serious unmet need for ARDS therapeutics that attenuate lung and systemic inflammatory injuries, reduce lung fluid imbalance, limit MOD and improve survival.

## Materials and Methods

### Reagents and Antibodies

Reagents unless specifically stated were obtained from Sigma-Aldrich (St. Louis, MO). Details of the eNAMPT mAb (ALT-100) have been previously reported (Sun et al., 2021; Garcia et al., 2022a; Bermudez et al., 2022) and was provided by Aqualung Therapeutics (Tucson, AZ). Western blot analyses details on antibodies utilized provided in Supplemental Methods. All assays and analyses were performed blindly to avoid selection or sampling biases. All analyses used a minimum of n = 4/group for the porcine study and n = 6/group for the rat study.

### Animals Studies

All experiments were approved by the Institutional Animal Care and Use Committee of University of Arizona and were performed in compliance with ARRIVE guidelines. All animals were blindly randomized to different groups to avoid any confounding variables. The laboratory manager was the only personnel aware of the animal treatment. All animals were housed under standard conditions and allowed to acclimatize for 5–7 days before the study. All animals used for this study were healthy naïve animals. See Supplemental Methods for additional details.

### Preclinical LPS/VILI Rat Model

Sprague Dawley (SD) male rats (250–300 gm, Charles River, Boston MA) (n = 6/group), were anesthetized and exposed to intratracheal LPS (0.1 mg/kg, 22 h) and mechanical ventilation for 4 h exactly as we have previously described (Mathew et al., 2011; Nonas et al., 2006; Sammani et al., 2010). The study groups were: control (n = 6), LPS/VILI with PBS treatment (n = 6), LPS/VILI with mAb treatment -4 h (n = 6), LPS/VILI with mAb treatment -8 h (n = 6), and LPS/VILI with mAb treatment -12 h (n = 6). See Supplemental Materials and Methods for additional details.

### Preclinical Pneumonia/Septic Shock/VILI Porcine Model

Yucatan male minipigs (17–20 kg, n = 4/group) were anesthetized and exposed to IV LPS (25 ug/kg, 2 h infusion) and to mechanical ventilation as we previously described (Bermudez et al., 2022). Pigs also received intratracheal LPS (50 ug/kg, 10 ml solution) via a bronchoscope. At either 2 h or 6 h post onset of LPS/VILI, pigs received either PBS or eNAMPT-neutralizing mAb (ALT-100, 0.4 mg/kg) (IV bolus 10 min). Animals were continuously monitored for mean arterial pressure (MAP), arterial blood gases (ABG), end tidal carbon dioxide (ETCO2) and both fluid input and output. To keep MAP >60 mmHg, IV infusion with a maintenance dose of 0.9% NaCl (10 ml/kg for the first hour) was increased when needed. If increased IV0.9% NaCl infusion fails to maintain the MAP >60 mmHg, vasopressors such as Norepinephrine and Phenylephrine were started. See Supplementary Table S4 and Supplementary Table S5 for measurements of P/F ratios and fluid administration throughout the 12 h of the experimental period in each animal group tested.

### Bronchoalveolar Lavage (BAL) Analysis

BAL studies in rats and pigs were performed exactly as previously described (Quijada et al., 2021; Bermudez et al., 2022). See Supplemental Methods.

### Chest Ultrasonography Analyses

Chest ultrasound images were obtained by a trained ultra-sonographer to detect and count the number of B-lines reflecting lung fluid accumulation (lung edema). Ultrasound in areas of dense lung consolidation precluded B-lines counting and were given a maximum B-line count of 20. See Supplemental Methods.

### Chest Radiograph Analyses

Inspiratory chest radiographs (Baseline 0 h and 12 h post-injury) were obtained from each pig using portable digital radiography. X-ray images in JPEG format were imported into ImageJ and lung field segmentation manually performed. Bimodal processing method was developed to fit a bimodal distribution to the histogram of the pixel gray scale values for each image. Each normal distribution is characterized by its mean (mu), and statistical differences in relative changes in normalized mu1 values grouped for 0 h and 12 h in treatment and non-treatment groups were analyzed (PBS, 2 h mAb, 6 h mAb). See Supplemental Methods.

### Lung Tissue Wet/Dry (W/D) Weight Ratios

Wet weight of samples was measured immediately after porcine lung tissue harvesting (upper, middle, lower lobes of right lung). Lung tissues were incubated in a 65°C vacuum oven for 72 h to obtain the dry weights with wet/dry weight ratios calculated to quantify the level of lung edema and fluid accumulation. See Supplemental Methods.

### Tissue Albumin Measurements

Homogenized lung tissue samples were diluted and assessed in a porcine-specific ELISA albumin kit. See Supplemental Methods.

### Quantitative Histology and Immunohistochemistry (IHC) Analyses

Rat and porcine tissues were collected for H&E histological assessment and IHC staining for NAMPT with ImageJ Quantification was performed as we previously described (Quijada et al., 2021; Bermudez et al., 2022). See Supplemental Methods.

### Plasma Biomarker Measurements

A meso-scale ELISA-based, U-PLEX linked platform was utilized (Meso Scale Diagnostics, Rockville, MD) for measurements of plasma levels of eNAMPT, IL-6, IL-8, and angiopoetin-2 as we previously described (Quijada et al., 2021; Bermudez et al., 2022). Rat and pig plasma samples were also assayed for Lipocalin-2, recognized AKI biomarker (Chueh et al., 2020; Yoo et al., 2020) using rat- and pig-specific Lipocalin-2 ELISA kits (Abcam, Cambridge, MA). See Supplemental Methods.

### Biochemical Analyses of Lung Homogenates

Western blotting of lung tissue proteins was performed with densitometric analysis normalized to β-actin expression as previously reported (Quijada et al., 2021). The levels of phosphor-proteins were quantified by normalizing the levels to their respective total proteins. See Supplemental Methods.

### Lung Tissue RNASeq Analysis

Total RNA was extracted from control and LPS/VILI-exposed rat and porcine lung tissues with RNA QC performed as previously reported (Ahmed et al., 2021; Garcia et al., 2022a; Garcia et al., 2022b) and RNA sequenced using the BGISEQ platform. DEseq2 (Cock et al., 2010) algorithms were used to detect differentially-expressed genes (DEGs). To control for multiple testing error, a false discovery rate (FDR) (Benjamini and Hochberg, 1995) was applied. Enriched analysis was conducted applying Gene Ontology (GO) classification, focused on biological process and pathway classification for the statistically-significant DEGs. Unbiased comparison of the gene sets to Consensus Path DB (Kamburov et al., 2013) against KEGG and Reactome pathways databases was conducted as previously reported (Ahmed et al., 2021; Garcia et al., 2022a; Garcia et al., 2022b). The STRING database was used to construct the interaction networks (Szklarczyk et al., 2017). See Supplemental Methods.

### Statistical Analyses

Continuous data were compared using nonparametric methods and categorical data by chi square test. Where applicable, standard one-way ANOVA was used, and groups were compared using the Newman-Keuls test. Differences between groups were considered statistically significant with *p* < 0.05. *t*-test was used to compare the means of data from different experimental groups. If significant differences were present by T-test (*p* < 0.05), a least significant differences test was performed post hoc. See Supplemental Methods for additional information.

## Results

### The eNAMPT-Neutralizing ALT-100 mAb Rescues Pneumonia/Sepsis/VILI-Induced Porcine Lung Injury

**FIGURE 1:**
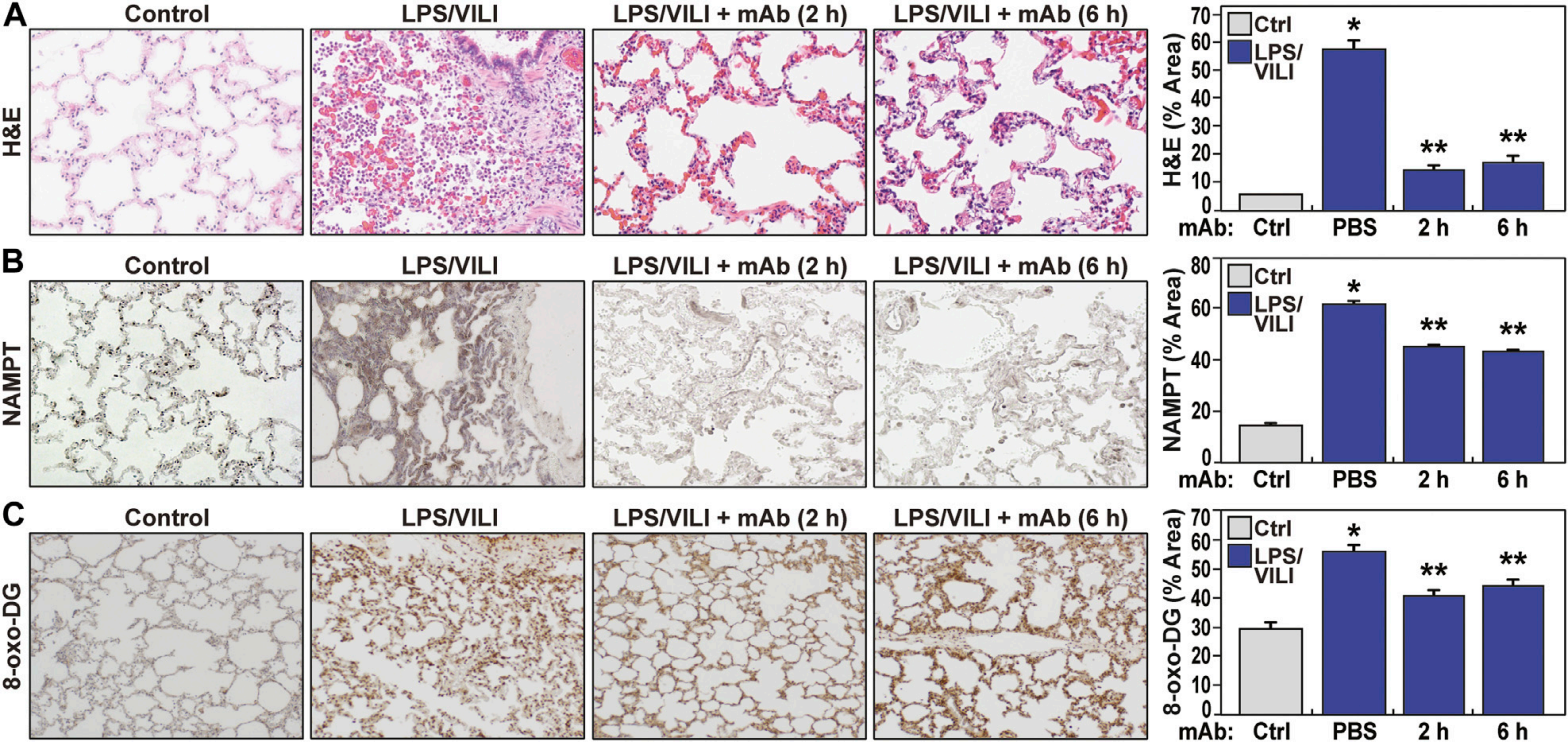
The eNAMPT ALT-100 mAb attenuates inflammatory lung injury in the LPS/VILI porcine model. **(A)** Representative H&E staining of lung tissues from Yucatan male minipigs (17–20 kg, n = 4/group) exposed to intratracheal LPS-induced pneumonia and IV LPS-induced septic shock accompanied by VILI for 12 h. Compared to control animals, LPS/VILI-exposed minipigs receiving IV PBS at 2 h exhibited dramatic histologic evidence of severe inflammatory lung injury with significant alveolar inflammation, neutrophil infiltration and alveolar edema quantified by Image J. The administration of IV ALT-100 eNAMPT mAb (0.4 mg/kg), delivered either 2 h or 6 h after the start of LPS/VILI injury, resulted in significantly reduced lung inflammation quantified by ImageJ (*p < 0.01 vs. control; **p < 0.01 vs. PBS). Magnification scale bar represents 50 uM. **(B)** Representative prominent increases in NAMPT lung tissue staining in LPS/VILI-exposed pigs detected by IHC staining. LPS/VILI-exposed minipigs receiving ALT-100 mAb intravenously at 2 h or 6 h exhibited significantly reduced NAMPT staining. **(C)** Similar to Panel B, representative 8-oxo-DG staining of lung tissues from LPS/VILI-exposed pigs showed prominent increases in 8-oxo-DG staining reflecting marked increases in reactive oxygen species (ROS) generation. LPS/VILI-exposed minipigs receiving IV ALT-100 mAb at 2 h or 6 h exhibited significantly reduced 8-oxo-DG staining. *p < 0.05 control vs. untreated LPS/VILI; **p < 0.05 mAb LPS/VILI vs. untreated LPS/VILI. There were no significant differences in imaging responses between animals receiving ALT-100 mAb at 2 vs. 6 h.

**FIGURE 2:**
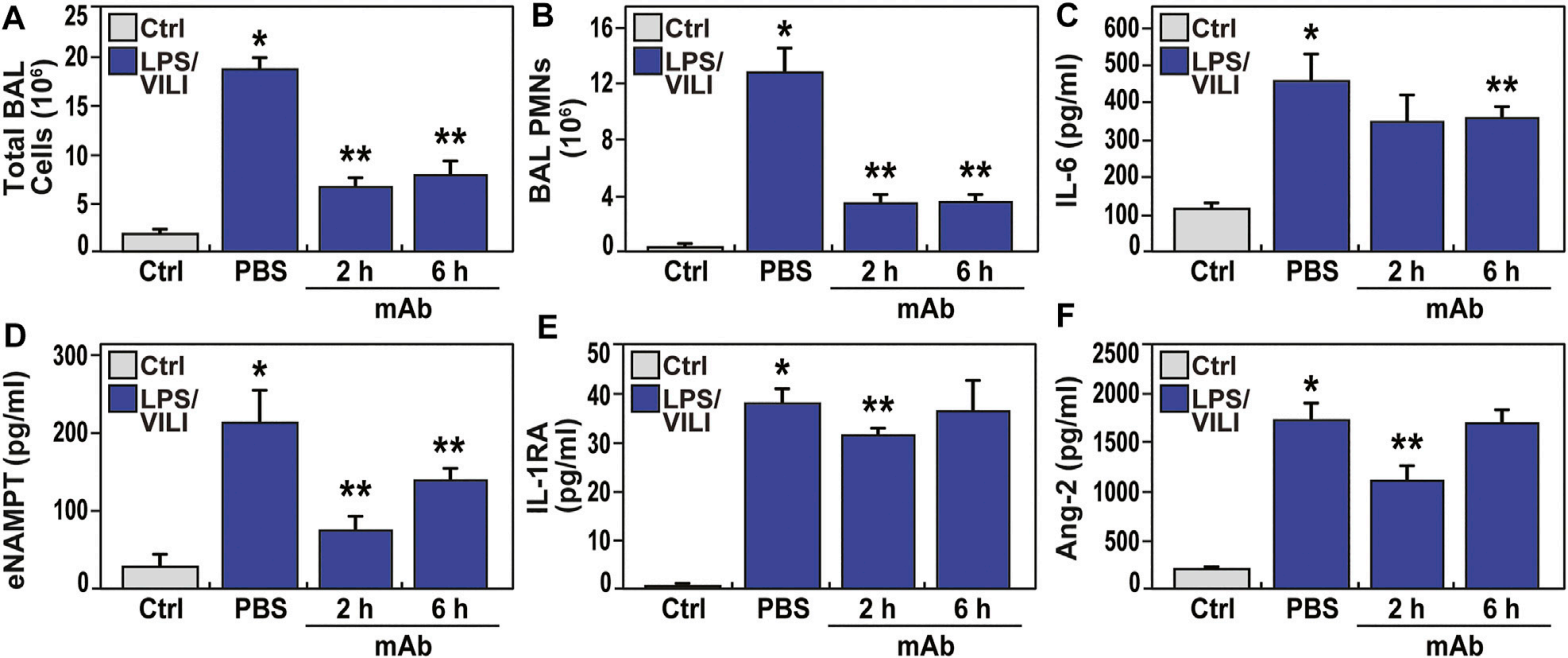
The eNAMPT-neutralizing ALT-100 mAb attenuates BAL alveolitis and plasma ARDS biomarker increases in the LPS/VILI porcine model. **(A,B)** Consistent with H&E findings (Figure 1), LPSVILI-exposed minipigs exhibit significant increases in BAL total inflammatory cell counts and BAL PMNs which are markedly attenuated in pigs receiving the ALT-100 mAb (0.4 mg/kg), delivered IV either 2 h or 6 h after initiation of LPS/VILI (*p < 0.05 vs. control, **p < 0.05 vs. untreated LPS/VILI). **(C–F)** Plasma levels of IL-6, eNAMPT, IL-1RA and angiopoetin-2 (Ang-2) measured by MesoScale Discovery platform at time 0 and at 12 h were markedly increased in LPS/VILI-challenged pigs. The levels of each biomarker were reduced in pigs receiving the ALT-100 mAb at 2 and 6 h after initiation of LPS/VILI. ALT-100 mAb-mediated reductions in eNAMPT and Ang-2 plasma levels were significantly greater in 2 h when compared to 6 h treated animals (*p < 0.05 vs. control, **p < 0.05 vs. untreated LPS/VILI).

Compared to controls, PBS-treated pigs exposed to 12 h of LPS/VILI revealed dramatic alveolar inflammation, neutrophil infiltration, and edema (Figure 1A), prominent increases in NAMPT tissue expression (Figure 1B) and increased 8-oxo-DG staining reflecting tissue reactive oxygen species (ROS) (Figure 1C). Lung tissue staining from pigs receiving the eNAMPT-neutralizing ALT-100 mAb (0.4 mg/kg) delivered IV either 2 h or 6 h after IT/IV LPS administration and VILI initiation, exhibited dramatic reductions in histologic inflammatory injury, NAMPT expression and ROS burden (Figure 1A–C), results confirmed by ImageJ analyses. ALT-100 mAb protection was comparable whether delivered IV at 2 h or 6 h after initial LPS/VILI exposure. Consistent with lung histologic findings, LPS/VILI exposure elicited significant elevations in total BAL cells/PMNs compared to PBS controls, which were reduced by ALT-100 mAb treatment at 2 h/6 h after onset of LPS/VILI injury (Figure 2A, B) as were the significant increases in plasma levels of IL-6, eNAMPT, IL-1RA and Ang-2 observed at 12 h (Figures 2C–F).

### The eNAMPT-Neutralizing ALT-100 mAb Preserves Lung Fluid Balance

**FIGURE 3:**
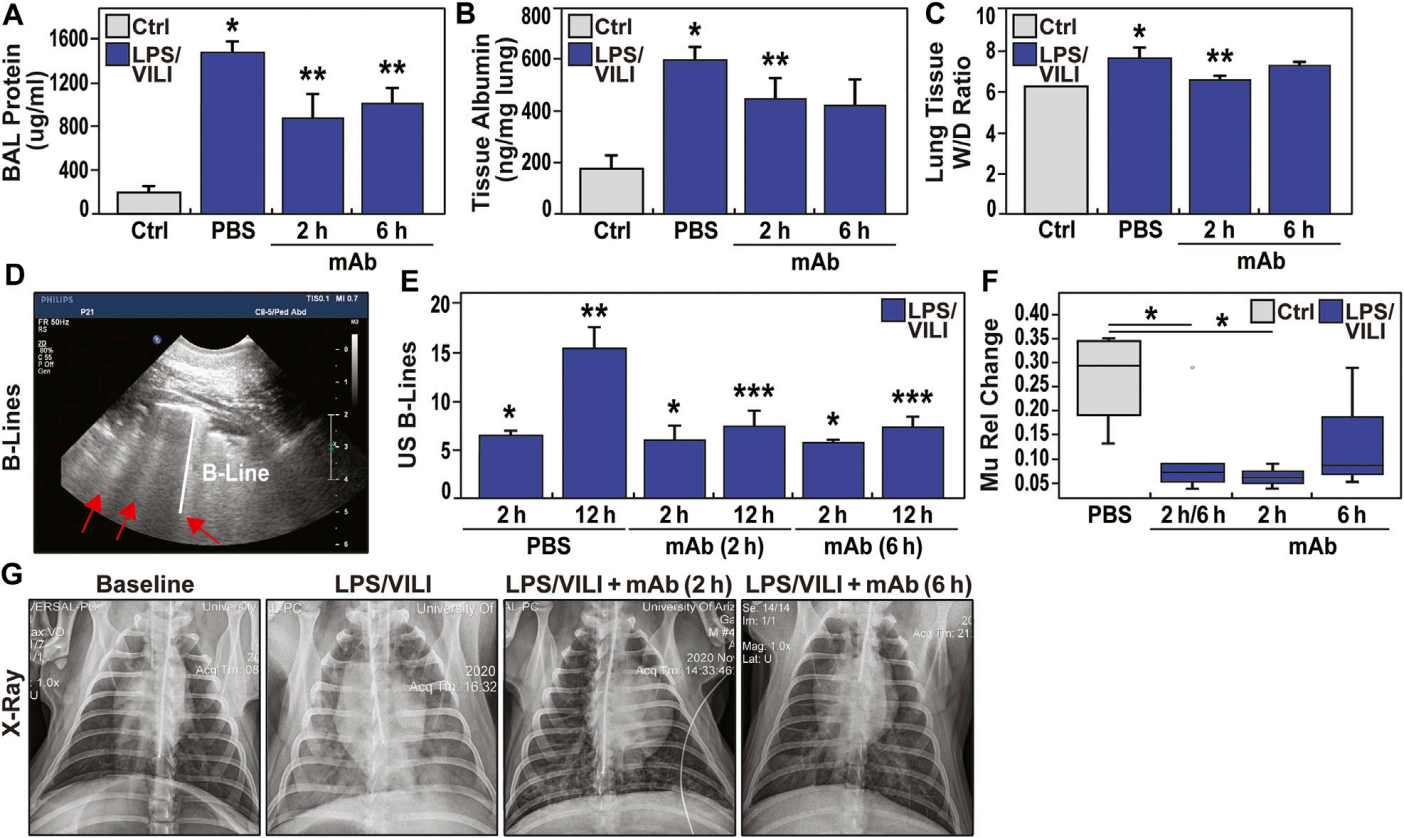
eNAMPT ALT-100 mAb attenuates lung edema formation in LPS/VILI porcine model. **(A,B)** LPS/VILI-exposed minipigs exhibit significantly increased levels of BAL total protein and lung tissue albumin levels at 12 h consistent with lung vascular and epithelial barrier dysfunction. Minipigs receiving ALT-100 mAb at either 2 h or 6 h demonstrated significant reductions in BAL protein and lung tissue albumin. *p < 0.05 control vs. untreated LPS/VILI; **p < 0.05 mAb LPS/VILI vs. untreated LPS/VILI. **(C)** Lung tissue wet/dry (W/D) weight ratios were calculated to quantify the level of lung edema and fluid accumulation as we previously described (Bermudez et al., 2022). These studies revealed significant W/D weight ratio increases in LPS/VILI-challenged pigs compared to control group which were significantly reduced in pigs receiving ALT-100 mAb at 2 h with pigs treated at 6 h trending toward significant reductions (7.22 ± 0.14 vs. 7.82 ± 0.43, *p* = 0.1). **(D,E)** Ultrasound-based measurements (4 lung quadrants) of the average number of B lines, a reflection of lung water/edema, were obtained. B lines were completely absent at baseline (not shown) but prominently increased in all 3 treatment groups (PBS, mAb 2 h, mAb 6 h) after 2 h of LPS/VILI exposure, further increasing when assessed at 12 h. Pigs treated with ALT-100 mAb (0.4 mg/kg) at either 2 h or 6 h post onset of injury, showed marked reductions in B lines at 12 h compared to the PBS-treated group (*p < 0.05 vs. control, uninjured pigs; **p < 0.05 baseline (BL) vs. 12 h endpoint in LPS/VILI-exposed pigs; ***p < 0.05 12 h mAb 2 and 12 h mAb 6 vs. 12 h PBS pigs). **(F,G)** Representative chest radiographs obtained at baseline (0 h, prior to LPS/VILI challenge) and after 12 h exposure to LPS/VILI with quantification of lung edema by measuring the change in mu1 Rel value at 12 h compared to its own 0 h baseline. The relative CXR mu1 values were markedly elevated at the 12 h endpoint in untreated LPS/VILI pigs. In contrast, mu1 values in the combined ALT-100 mAb treated pigs at 2 and 6 h were significantly diminished when compared to untreated LPS/VILI pigs (*p* = 0.03). This reduction remained significant when only 2 h pig mu1 values were considered (*p* = 0.02). The mu1 values from ALT-100 6 hrs-treated pigs were not significantly different compared to untreated LPS/VILI pigs (*p* = 0.2).

Increases in lung fluid imbalance secondary to unremitting vascular permeability is a major driver of MOD and ARDS mortality. Figure 3A–C depicts the multi-pronged approach to assess lung fluid imbalance in LPS/VILI-exposed pigs with significant increases in BAL total protein levels, lung tissue albumin measurements, and lung tissue wet/dry (W/D) weight ratio measurements consistent with lung vascular and epithelial barrier dysfunction. In addition, we utilized an ultrasound-based readout of LPS/VILI-induced lung edema with B-line quantification as a quantitative reflection of lung edema. B-lines were entirely absent at baseline and in control animals but dramatically increased at 2 h of LPS/VILI exposure, with further significant increases at 12 h (Figure 3D, E). We also utilized chest radiograph (CXR) pixel histogram quantification of the segmented lungs to reflect increased fluid accumulation in injured lungs at 12 h (increased mu1 values 0 h to 12 h) (Figure 3F, G). For each lung fluid imbalance readout, pigs receiving the eNAMPT-neutralizing ALT-100 mAb, either at 2 h or 6 h after LPS/VILI injury, demonstrated significant preservation of lung fluid balance compared to PBS-treated, LPS/VILI-exposed pigs. Lung fluid balance was comparable for ALT-100 mAb delivered at 2 vs. 6 h with the exception of lung tissue albumin levels and CXR mu1 values which were significantly better preserved in pigs treated at 2 h.

### The eNAMPT-Neutralizing ALT-100 mAb Reduces LPS/VILI-Induced Acute Kidney Injury

**FIGURE 4:**
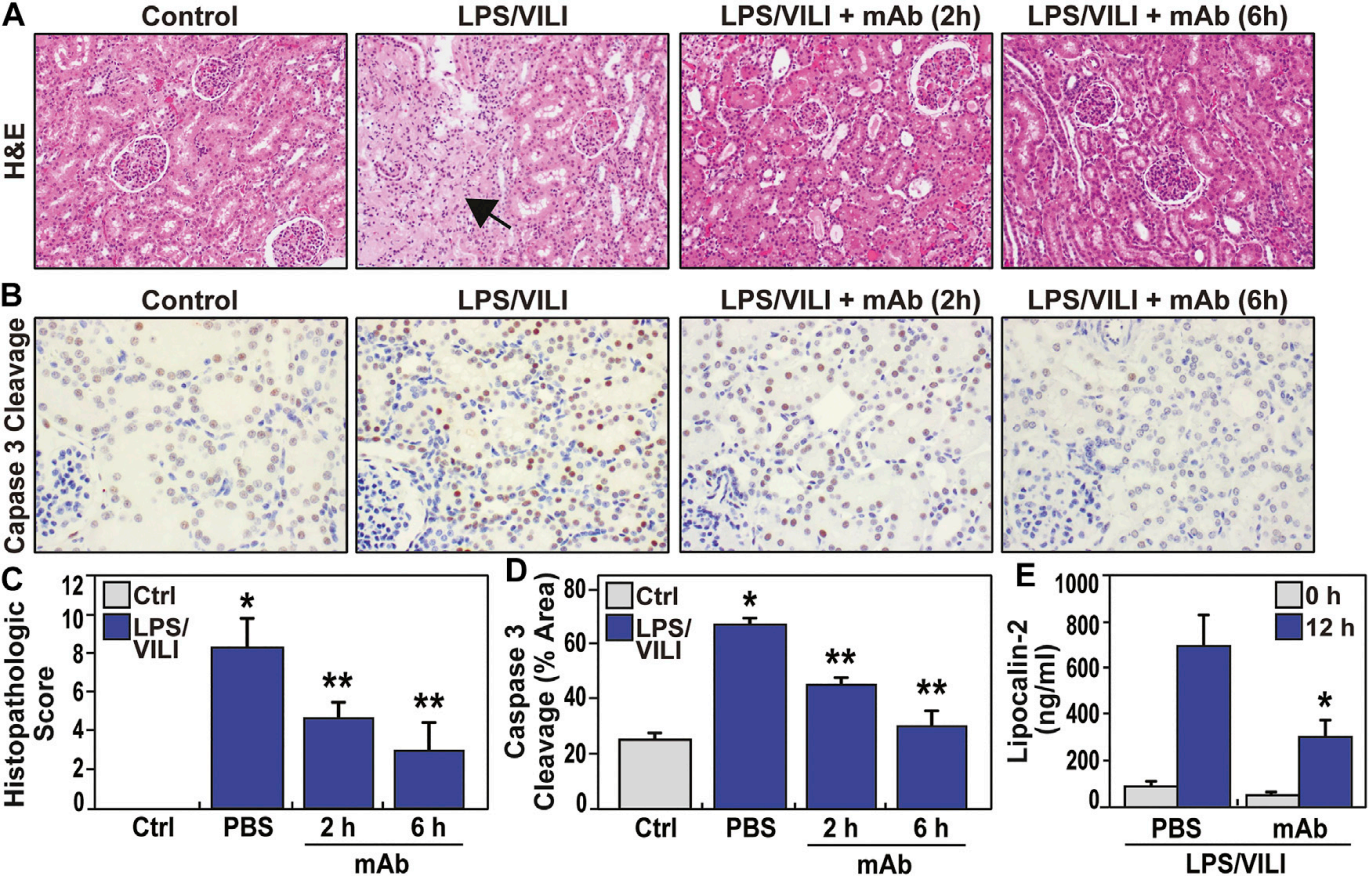
eNAMPT ALT-100 mAb treatment attenuates acute kidney injury (AKI) in LPS/VILI porcine model. **(A,C)** Representative H&E renal histology in LPS/VILI-exposed minipigs showing marked tubular duct dilation accompanied by areas of frank parenchymal necrosis (arrow) compared to control, unchallenged pigs. Animals receiving the ALT-100 mAb (2 h or 6 h after injury initiation) exhibited dramatic preservation of tissue architecture with minimal evidence of renal injury (*p < 0.05 control vs. untreated LPS/VILI; **p < 0.05 mAb LPS/VILI vs. untreated LPS/VILI). **(B,D)** Representative IHC image of cleaved capase-3 (CC3, marker of apoptosis) in renal tissues from PBS- and ALT-100 mAb-treated pigs at 12 h showing prominent increases in apoptosis in renal tissues. Pigs receiving the eNAMPT mAb (2 h or 6 h after initiation) showed dramatically reduced caspase 3 cleavage staining. **(E)** LPS/VILI exposure significantly increases plasma lipocalin levels at 12 h (AKI marker) which are significantly reduced in ALT-100 mAb-treated pigs (2 and 6 h combined).

Acute kidney injury (AKI) is the most common vital organ to fail in ARDS (Banoth et al., 2015; Peerapornratana et al., 2019). Laboratory assessment of renal indices (BUN, creatinine) showed a trend toward renal impairment in LPS/VILI-exposed pigs with doubling of the serum creatinine, however, this was not statistically significant (Supplementary Table S1). In contrast, histologic assessment (H&E staining) of renal tissues from LPS/VILI-exposed pigs at 12 h showed prominent AKI with renal tubular dilatation and glomerular necrosis as well as evidence of increased neutrophil and monocyte renal infiltration (Figure 4A, C). Histologic renal injury **was** markedly reduced in LPS/VILI pigs receiving the ALT-100 mAb at either 2 h or 6 h post injury onset, approaching normal renal histology (Figure 4A, C). IHC renal staining for cleaved caspase 3 (marker of inflammatory injury) showed in LPS/VILI-mediated increased staining which was markedly reduced in eNAMPT ALT-100-treated pigs (Figure 4B, D). Plasma lipocalin-2 levels, a well-recognized AKI biomarker, are markedly increased in LPS/VILI-exposed pigs. Consistent with eNAMPT mAb-mediated reductions in histologic renal injury, plasma lipocalin-2 levels were reduced in pigs receiving ALT-100 mAb (Figure 4E).

### The eNAMPT ALT-100 mAb Rectifies Dysregulated Inflammatory Signaling in LPS/VILI-Induced Porcine Lung Injury

**FIGURE 5:**
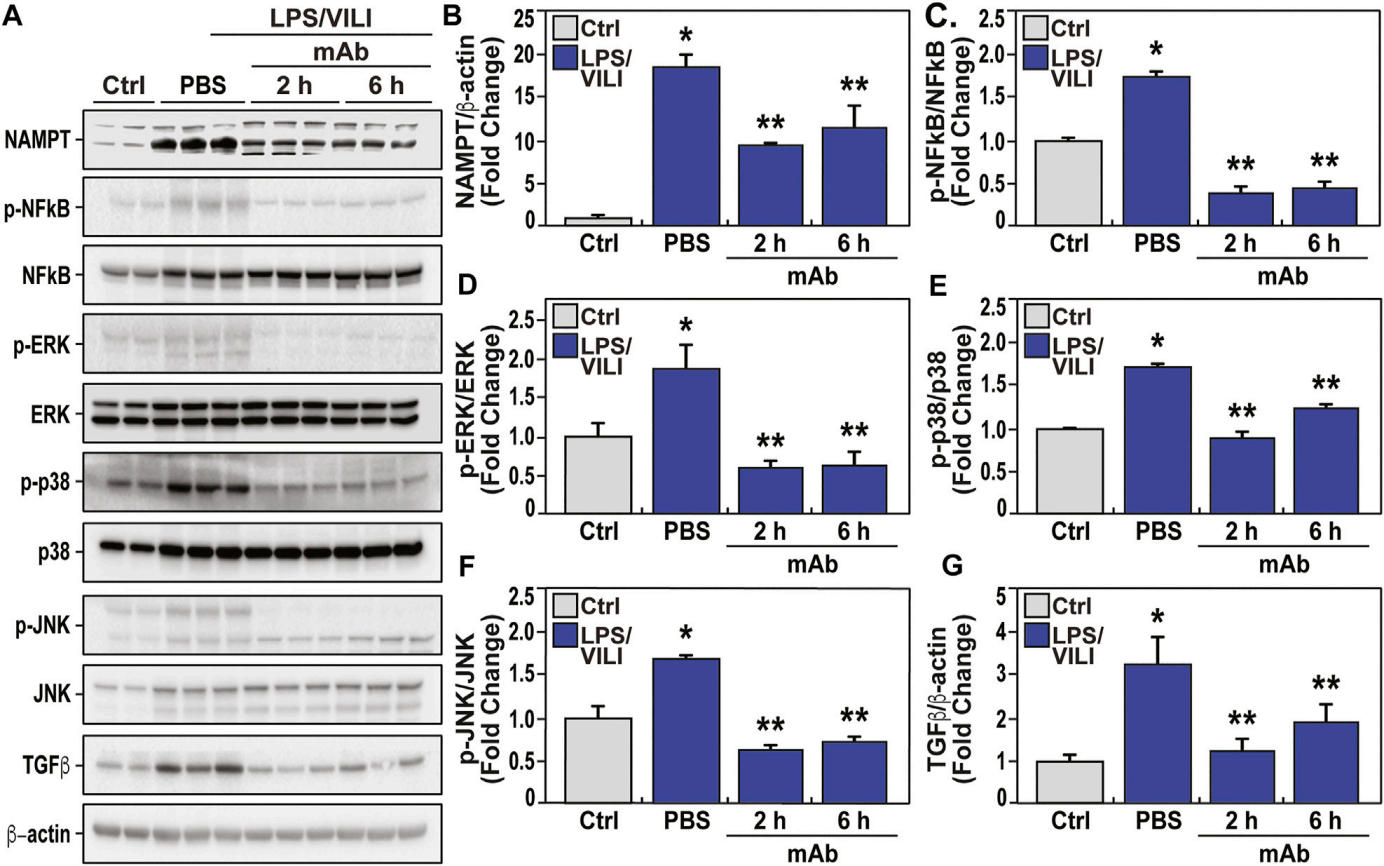
The eNAMPT-neutralizing ALT-100 mAb attenuates dysregulated NFkB and MAP kinase signaling in an LPS/VILI porcine model. Lung tissue homogenates were obtained from PBS-treated and eNAMPT ALT-100 mAb-treated LPS/VILI-exposed pigs (n = 4/group) and compared to untreated control pigs (n = 4). **(A,B)** Western blot analysis confirmed significantly higher NAMPT expression in porcine LPS/VILI lung homogenates (lanes 3–5) compared to controls (first 2 lanes). The eNAMPT-neutralizing ALT-100 mAb reduced NAMPT immunoreactivity in lung homogenates both when delivered at 2 h (lanes 6–8) and at 6 h (lanes 9–11) (*p < 0.05 control vs. untreated LPS/VILI; **p < 0.05 mAb LPS/VILI vs. untreated LPS/VILI). **(A,C)** Western blotting studies revealed striking increases in NFkB phosphorylation/total NFkB levels in LPS/VILI porcine tissues which were abolished by ALT-100 mAb when delivered at 2 h (lanes 6–8) and at 6 h (lanes 9–11) confirmed by densitometric evaluation of the ratio of p-NFkB/NFkB shown in the bar graph (*p < 0.05 control vs. untreated LPS/VILI; **p < 0.05 mAb LPS/VILI vs. untreated LPS/VILI). **(A,D,E,F)** Western blotting studies of LPS/VILI-exposed lung homogenates detected prominent MAP kinase family activation with increased levels of ERK, p38 and JNK phosphorylation (pp-42/44 ERK, pp-p38, pp-JNK). MAP kinase family activation which was attenuated in pigs receiving the ALT-100 mAb delivered at 2 h (lanes 6–8) or 6 h (lanes 9–11) (*p < 0.05 control vs. untreated LPS/VILI; **p < 0.05 mAb LPS/VILI vs. untreated LPS/VILI. **(A,G)** Total TGF^ꞵ^protein levels in LPS/VILI lung homogenates were markedly increased and reduced in pigs receiving the eNAMPT-neutralizing ALT0-100 mAb shown by densitometric assessment (*p < 0.05 control vs. untreated LPS/VILI; **p < 0.05 mAb sepsis/VILI vs. untreated LPS/VILI).

Biochemical analyses of LPS/VILI-exposed lung tissues identified striking increases in NAMPT expression (Figure 5A, B) and in levels of phosphorylated NFkB (Figure 5A, C) and MAP kinases (ERK, p38, JNK) (Figure 5A, D, E, F), and significant increases in TGFβ expression, reflecting strong activation of these inflammatory signaling pathways. Dysregulation of each inflammatory signaling pathway (eNAMPT, NFκB, MAP kinase, TGF) was markedly attenuated in pigs receiving the eNAMPT-neutralizing ALT-100 mAb (2 h, 6 h) (Figure 5). These studies are highly consistent with a critical role for the eNAMPT/TLR4 signaling pathway in LPS/VILI-induced activation of evolutionarily-conserved inflammatory cascades that contribute to ARDS pathobiology, severity and mortality (Gong et al., 2020; Bime et al., 2021).

### The eNAMPT-Neutralizing ALT-100 mAb Rescues LPS/VILI-Induced Rat Lung Injury

**FIGURE 6:**
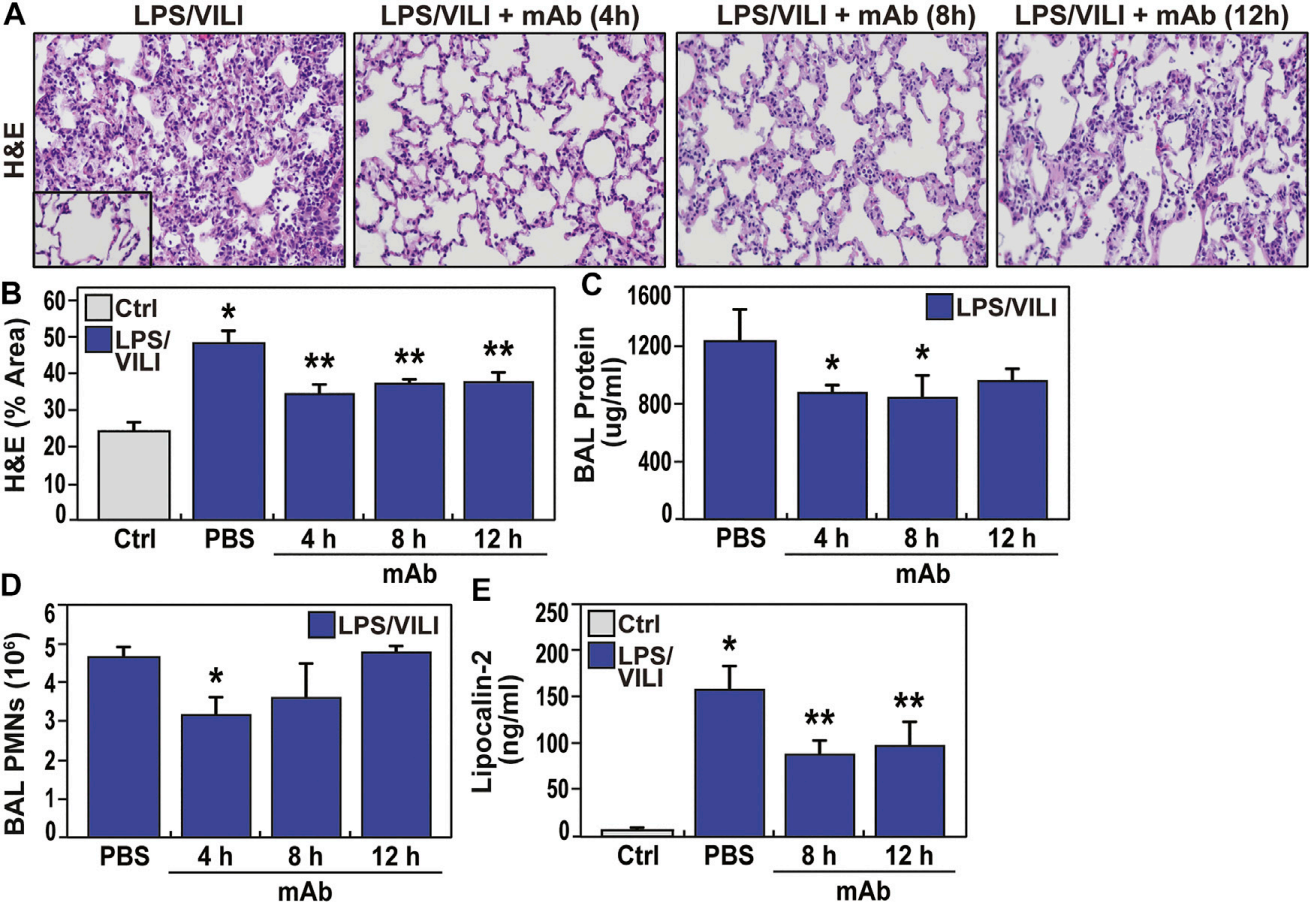
Delayed delivery of the ALT-100 mAb reduces inflammatory lung injury in a LPS/VILI-exposed rat model. **(A,B)** The capacity for the ALT-100 mAb to rescue from LPS/VILI-induced lung injury was assessed in Sprague Dawley rats (SD) rats (250–300 gm, n = 6/group) with the eNAMPT-neutralizing ALT-100 mAb (1 mg/kg) delivered at 3 time points post intratracheal LPS challenge (4, 8, and 12 h) prior to VILI (18–22 h). All animals were sacrificed after 22 h of LPS exposure and 4 h of mechanical ventilation. Compared to animals receiving PBS at time 0, rats receiving ALT-100 mAb (1 mg/kg) at either 4, 8, or 12 h post LPS injection, showed significant reductions in lung tissue H&E evidence of alveolar inflammation and edema (*p < 0.05 control vs. untreated sepsis/VILI; **p < 0.05 mAb LPS/VILI rats vs. untreated LPS/VILI rats). **(C,D)** Evaluation of BAL protein and BAL PMNs generation in LPS/VILI-exposed rats show marked increases in both inflammatory parameters which were reduced in rats receiving the eNAMPT-neutralizing ALT-100 mAb (1 mg/kg), with maximal reduction protection when delivered at the earliest 4 h time point after LPS exposure (*p < 0.05). **(E)** Similar to studies in LPS/VILI-exposed pigs, LPS/VILI-exposed rats show significant increases in plasma lipocalin levels at 22 h (AKI marker) which are significantly reduced in ALT-100 mAb-treated rats at 8 and 12 h post LPS injection (*p < 0.05 control vs. untreated LPS/VILI rats; **p < 0.05 mAb LPS/VILI rats vs. untreated LPS/VILI rats).

To corroborate ALT-100 mAb rescue in LPS/VILI-exposed pigs, we assessed the utility of delayed delivery of ALT-100 mAb in LPS/VILI-exposed rats. Figures 6A, B depicts the significant protective effect of ALT-100 mAb (1 mg/kg) on LPS/VILI-induced histologic lung injury when given at 4, 8, and 12 h after LPS injection (prior to VILI exposure). These data show reduced histologic alveolar inflammation and neutrophil infiltration (Figure 6A, B) and reduced BAL protein and PMNs (Figure 6C, D), even in animal receiving eNAMPT ALT-100 mAb at 12 h post LPS exposure. In addition, plasma lipocalin levels in LPS/VILI-exposed rats reflecting AKI were attenuated by ALT-100 mAb delivered 8–12 h after LPS exposure (Figure 6E).

### The eNAMPT ALT-100 mAb Rectifies LPS/VILI-Induced Gene Dysregulation in Rat and Porcine Lung Tissues

**FIGURE 7:**
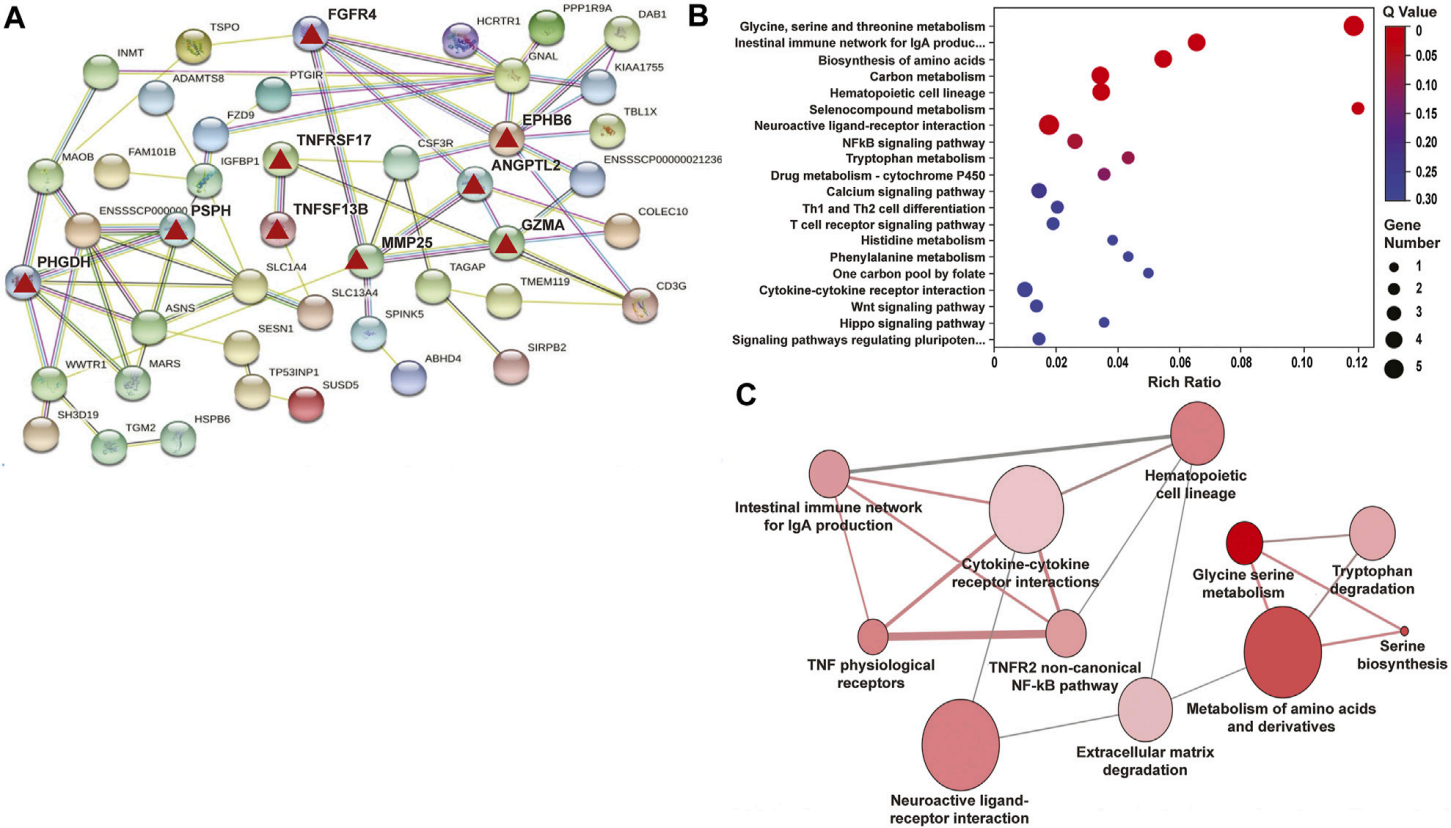
Differentially-expressed genes (DEGs)/pathways in LPS/VILI-exposed porcine lung tissues: Influence of ALT-100 mAb on gene network interactions. RNA sequencing data of lung tissues yielded 332 differentially-expressed genes (DEGs) between control pigs and LPS/VILI-challenged pigs (12 h) (Table 1) and 465 DEGs from LPS/VILI-exposed rats (Supplementary Table S1). The # of DEGs influenced by ALT-100 mAb in from LPS/VILI-challenged pigs is 62 (Table 2) and in rats is 34 (Table 3). **(A)** Shown is the STRING Functional protein network interaction of DEGs influenced by ALT-100 mAb treatment in pigs. The edges represent protein-protein associations, line color indicates the evidence the type of interaction (green-gene neighborhood, red-gene fusion, blue-co-occurrence, black-co-expression). The confidence interaction score is 0.15. Highlighted are specific genes (red triangles) that are detailed in the Discussion. **(B,C)** Shown are the top 20 KEGG enriched pathways in the ALT-100 mAb rescued porcine model. The Rich factor is the ratio of DEGs numbers annotated in this pathway term to all gene numbers annotated in this pathway term. Q value is the corrected *p* value, the size of the bubble corresponds to the number of genes annotated in that pathway.

RNA sequencing data from LPS/VILI-exposed pigs further validated the mechanism of action for ALT-100 mAb efficacy as to involve dampening of inflammatory processes and cascades. Analyses of differentially-expressed genes (DEGs) between control pigs and LPS/VILI-challenged pigs (12 h) identified 332 DEGs (FDR 0.05, FC 2.5) that participate in inflammatory pathways such as cytokine-cytokine interactions, cell-adhesion molecules, chemokine signaling, cAMP signaling, MAP kinase signaling and PI3K/AKT signaling (Table 1). Comparisons of DEGs derived from PBS vs. ALT-100 mAb-treated LPS/VILI-challenged pigs identified 62 DEGs (FDR <0.1) again involved in inflammatory pathways such as TNF receptors (*TNFSF17, TNFSF13B*)*, IL12B*, angiopoietin-2, metalloproteinase 25, and genes associated with lung remodeling (*ADAMTS8, FGFR4*) depicted in a porcine STRING-based interactome (Figure 7A). Top prioritized KEGG pathways included prominent inflammation-related pathways such as glycine/serine/threonine metabolism (*MAOB; PHGDH; SHMT1; PSPH*), NFkB signaling, and cytokine-cytokine interactions (Figure 7B). TNF binding to their physiological receptors and TNFR2 non-canonical NF-kB pathways were among top Integrating Reactome-enriched pathways (Table 2). Non-canonical NFkB activation is generally stimulated by ligands of the TNF receptor superfamily that induces NFκB-inducing kinase (NIK), which leads to nuclear translocation of RelB-p52 heterodimer (Sun, 2017). Canonical NFκB activation relies on activation of IKK-mediated IκBα phosphorylation, and subsequent degradation, leading to nuclear translocation of NFκB heterodimer RelA(p65)/p50 (Hayden and Ghosh, 2008). TLR4 activation triggers canonical NF-KB activation to mediate inflammatory responses (Kawai and Akira, 2007). Recent reports suggest a possibility that non-canonical NF-kB activation contributes to infectious inflammation (Banoth et al., 2015; Sun, 2017). Our data raise a potential implication of the TNFR2 non-canonical NF-kB pathway in lung inflammatory injury, a pathway which is also attenuated by eNAMPT neutralization.

**TABLE 1:** Top DEG KEGG Pathways from LPS/VILI-exposed pigs versus controls.

KEGG pathway term desc	Candidates contained	Total gene number	*p* value	Q value
Cytokine-cytokine receptor interaction	175	296	8.44E-24	2.12E-21
Cell adhesion molecules (CAMs)	90	150	2.6E-13	3.27E-11
Axon guidance	101	179	1.97E-12	1.5E-10
Complement and coagulation cascades	57	83	2.4E-12	1.5E-10
Hematopoietic cell lineage	69	115	1.63E-10	8.19E-09
ECM-receptor interaction	53	82	4.71E-10	1.97E-08
Neuroactive ligand-receptor interaction	134	279	2.26E-09	8.11E-08
Calcium signaling pathway	104	204	2.65E-09	8.31E-08
Arachidonic acid metabolism	48	80	1.01E-07	2.81E-06
Phagosome	82	164	3.74E-07	9.4E-06
cGMP-PKG signaling pathway	82	165	5.19E-07	1.08E-05
cAMP signaling pathway	100	211	5.6E-07	1.08E-05
Melanogenesis	57	104	5.07E-07	1.08E-05
Chemokine signaling pathway	86	177	9.44E-07	1.69E-05
Steroid hormone biosynthesis	37	60	1.14E-06	1.87E-05
Vascular smooth muscle contraction	64	123	1.22E-06	1.87E-05
Pancreatic secretion	51	92	1.27E-06	1.87E-05
Drug metabolism - cytochrome P450	35	56	1.41E-06	1.93E-05
Insulin secretion	47	83	1.46E-06	1.93E-05
Ovarian steroidogenesis	33	52	1.72E-06	2.15E-05
MAPK signaling pathway	131	298	2.03E-06	2.42E-05
PI3K-Akt signaling pathway	154	361	2.27E-06	2.59E-05

**TABLE 2:** Top DEG Pathways in LPS/VILI-exposed pigs: ALT-100 mAb-treated versus PBS.

Pathway	*p* value	Q value	Source	DEG names	Effective size
Glycine, serine, and threonine metabolism	4.62E-06	2.63E-04	KEGG	*MAOB; PHGDH; SHMT1; PSPH*	40
Serine biosynthesis	2.77E-04	6.55E-03	Reactome	*PHGDH; PSPH*	9
Metabolism of amino acids and derivatives	3.45E-04	6.55E-03	Reactome	*INMT; PSPH; ASNS; CRYM; PHGDH; SHMT1*	339
Neuroactive ligand-receptor interaction	2.58E-03	2.86E-02	KEGG	*HCRTR1; TSPO; APLNR; GZMA; PTGIR*	341
Hematopoietic cell lineag**e**	2.64E-03	2.86E-02	KEGG	*CD37; CSF3R; CD3G*	98
TNFs bind their physiological receptors	3.01E-03	2.86E-02	Reactome	*TNFRSF17; TNFSF13B*	29
Tryptophan metabolism -	6.25E-03	5.09E-02	KEGG	*MAOB; INMT*	42
Intestinal immune network for IgA production - (human)	7.77E-03	5.54E-02	KEGG	*TNFRSF17; TNFSF13B*	47
TNFR2 non-canonical NF-kB pathway	8.76E-03	5.55E-02	Reactome	*TNFRSF17; TNFSF13B*	50
NF-kappa B signaling pathway	8.64E-03	6.81E-02	KEGG	*TNFSF13B, LOC100621559, LOC110258822*	114

Comparison of control rats to LPS/VILI-challenged rats, identified 465 DEGs (FDR 0.05, FC 2.5) involved in inflammatory pathways such as cytokine-cytokine receptor interaction, complement and coagulation cascades, IL-17 signaling, and metabolic-related pathways (Supplementary Table S2). We next compared LPS/VILI-challenged pigs/rats treated with ALT-100 mAb vs. PBS and identified 34 DEGs (FDR 0.1) including *Igr1r, cd36;* and *adipoq* which are enriched for AMPK signaling pathways, and adipocytokine and PPAR signaling pathways **(** Supplementary Figure S1, Table 3). These DEGs were used to generate a STRING-based rat interactome with *Igfr* (insulin-like receptor), *Adipoq* (adipokine), and *DII1,* a NOTCH receptor, as the highest DEGs identified.

**TABLE 3:** Top DEG Pathways in LPS/VILI-exposed rats: ALT-100 mAb-treated versus PBS.

Pathway	*p* value	Q value	Source	DEG names	Effective size
AMPK signaling pathway	6.07E-04	1.40E-02	KEGG	*IGF1R; CD36; ADIPOQ*	120
Adipocytokine signaling pathway	4.20E-03	2.76E-02	KEGG	*CD36; ADIPOQ*	69
PPAR signaling pathway	4.82E-03	2.76E-02	KEGG	*CD36; ADIPOQ*	74
ECM-receptor interaction	6.75E-03	2.76E-02	KEGG	*IBSP; CD36*	88
Longevity regulating pathway	6.90E-03	2.76E-02	KEGG	*IGF1R; ADIPOQ*	89
Integration of energy metabolism	7.20E-03	2.76E-02	Reactome	*ADIPOQ; CD36*	91
Breast cancer	1.80E-02	5.92E-02	KEGG	*DLL1; IGF1R*	147
Wnt signaling pathway	2.26E-02	6.50E-02	KEGG	*BAMBI; FOSL1*	166
Focal adhesion	3.22E-02	8.24E-02	KEGG	*IGF1R; IBSP*	201
SLC-mediated transmembrane transport	4.55E-02	1.05E-01	Reactome	*SLC29A4; SLC22A3*	243

Finally, merging ALT-100 mAb-rescued DEGs in pigs and rats, identified common related pathways for both species (Supplementary Table S3). Furthermore, ortholog crossmatching of rat and pig LPS/VILI DEGs with human genes revealed 26 genes related to extracellular matrix organization (*MMP7, MMP8, MMP9, ADAMST4*), Toll-like receptor signaling (*CXCL11, CXCL9, SPP1*), and dysregulated immune-related pathways (Th1/Th2 cell differentiation, WTN signaling, MAPK signaling). Overall, these studies are consistent with an important role for the eNAMPT/TLR4 pathway in triggering LPS/VILI-induced inflammatory cascades that contribute to the severity of ARDS/VILI.

## Discussion

The current study was designed to directly address three essential but unmet needs in subjects with ARDS/VILI: 1) the need for a large animal ARDS/VILI model that recapitulates features of human ARDS/VILI thereby allowing for rigorous testing of novel therapeutics; 2) the need for novel phenotypic tools to fully assess preclinical lung fluid imbalance and multi-organ dysfunction, responses directly related to unchecked vascular permeability and ARDS mortality; and, 3) the need for novel therapeutics that reduce the severity of ARDS, MOF and mortality. The *first* unmet need highlights the preponderance of preclinical rodent ARDS models that utilize bacterial or LPS challenge models without concomitant exposure to VILI. We speculate that the bacteria/LPS-only models fail to sufficiently simulate clinical ARDS potentially contributing to the failed transition of promising therapies in rodents to successful human clinical trials. In contrast, our Yucatan minipig ARDS/VILI model exhibits a number of key features of human ARDS in ICU subjects including the capacity to assess the effects of prolonged mechanical ventilation (12 h VILI) on altered respiratory mechanics and compliance (Bermudez et al., 2022), lactic acidosis (Bermudez et al., 2022), and extrapulmonary organ injury (Figure 4), features not easily assessed in small rodent models where the maximal duration of ventilator exposure is ∼4–5 h.

In addition to allowing assessment of key pulmonary physiologic parameters (Bermudez et al., 2022), the porcine model is highly useful in directly addressing the *second* unmet ARDS need for novel phenotypic tools to assess the reversibility of lung fluid imbalance, a key pathophysiologic feature driving mortality in ARDS. Historically, this has been an exceptionally elusive parameter when assessed clinically via lung auscultation and standard CXRs which suffer from such low specificity and sensitivity to preclude tracking of even daily improvements in lung water balance. We utilized multiple complementary approaches to evaluate lung fluid balance including conventional preclinical measurements of BAL protein, lung tissue albumin content, and lung tissue wet/dry weight ratios. In addition, we report both ultrasonography-derived B lines and digital CXR analysis combined with pixel histogram quantification as useful tools to examine and quantify lung edema.

These tools allowed us to address the *third* unmet ARDS need for novel therapeutics that reduce MOF and ARDS/VILI severity. Our results provide additional supportive evidence that the humanized eNAMPT-neutralizing biologic, ALT-100 mAb is highly effective in attenuating the magnitude of eNAMPT/TLR4 inflammatory cascade activation in the combined ARDS and VILI porcine model (Quijada et al., 2021; Bermudez et al., 2022). Pigs receiving the ALT-100 mAb exhibited consistent reductions in each readout of LPS/VILI-induced lung edema and acute kidney injury (AKI), often the initial non-pulmonary organ affected in the course of ARDS and sepsis (Chueh et al., 2020; Yoo et al., 2020). and a key prognostic factor for mortality (Peerapornratana et al., 2019). LPS/VILI-induced AKI, reflected by histologic injury and increased blood levels of the AKI biomarker lipocalin, were significantly attenuated in pigs receiving ALT-100 mAb either at 2 h or 6 h after onset of injury or in rats receiving ALT-100 mAb up to 12 h after LPS.

Another important aspect of our study was the examination of the capacity for ALT-100 mAb to preserve lung fluid balance and minimize multi-organ dysfunction when delivered to LPS/VILI-exposed rats and minipigs with established inflammatory lung injury. Comparisons of multiple inflammatory indices showed trending toward greater protection with delivery of ALT-100 mAb at 2 vs. 6 h after onset of LPS/VILI exposure in minipigs. However, 2h mAb-mediated protection was only statistically significant for plasma eNAMPT and Ang-2 levels indicating that delayed ALT-100 mAb delivery, even up to 6 h after the onset of ARDS/VILI, significantly reduces inflammatory cascade activation. These findings were corroborated in rescue studies in LPS/VILI-challenged rats (Figure 6) with significant ALT-100 mAb-mediated lung and renal protection when delivered up to 12 h after LPS injection. These ALT-100 mAb rescue properties are critical to the clinical utility of this biologic as an ARDS therapeutic.

Our biochemical and genomic studies strongly confirmed the mechanism of action for ALT-100 mAb is via robust dampening of eNAMPT/TLR4 inflammatory cascade activation thereby rectifying LPS/VILI-dysregulated genes, proteins, and pathways that drive the severity of inflammatory injury. These include well-recognized inflammatory receptors such as Toll-like receptors, TNFα, and TGFβ, and downstream signaling by NFkB, MAP family kinases (ERK, JNK, p38), and PI3K-Akt signaling pathways (Bermudez et al., 2022). Examination of the porcine STRING Interactome of mAb-influenced genes (Figure 7A) revealed strong thematic rectification of ARDS inflammation targets/effectors including *Ang-2* (Angiopoietin 2, a validated ARDS biomarker) (Bime et al., 2019; Bime et al., 2021), *MMP25* (metalloprotease 25, an innate immunity regulator) (Soria-Valles et al., 2016), *GNAL* (a G protein subunit involved in phospholipase C and ERK signaling), *EphB6* (a kinase-dead EphB receptor involved in vascular inflammatory barrier responses (Coulthard et al., 2012)) and *GZMA* or granzyme A, a well-known inducer cell death and regulator of inflammatory cytokine production (van Daalen et al., 2020). The porcine Interactome also revealed an interesting pair of mAb-influenced DEGs (*PHGDH, PSPH*) which were also observed in the top dysregulated KEGG Term ‘serine/glycine/threonine metabolism’. *PHGDH* (3-phosphoglycerate dehydrogenase) and *PSPH* (phosphoserine phosphatase), are key enzymes involved in the *de novo* serine/glycine biosynthesis involved in suppression of cytokine production and mitochondrial dysfunction (Kurita et al., 2021).

The examination of the rat STRING Interactome revealed a single major hub gene/protein, *IGF1R* (Insulin-like Growth Factor 1 Receptor) (Supplementary Figure S2) which is influenced by the eNAMPT mAb. IGF1R is a pro-inflammatory tyrosine kinase involved in viral- and non-viral-induced inflammatory processes and cytokine secretion (Li et al., 2019) via PI3K/AKT and MAPK signaling pathways that are directly rectified by the ALT-100 mAb (Figure 6) (Bermudez et al., 2022). An Interactome protein, *ADIPOQ* or adiponectin (Robinson et al., 2011), similar to eNAMPT (aka visfatin), is a pro-inflammatory adipokine (Robinson et al., 2011; Dakroub et al., 2021; Mitsis et al., 2022) and known ARDS candidate gene/protein (Ahasic et al., 2014). Adiponectin is a prominent member of the “AMPK/NFκB pathway” (top KEGG Term pathway) that contributes to metabolic/bioenergetic alterations in sepsis-mediated organ injury (Liu et al., 2016).

The pathogenesis of ARDS is characterized by heterogeneous/multifactorial etiologies that initiate lung injury. Our model focuses on the LPS/VILI injury which is the common etiology but a limitation of our study as it does not address the effectiveness of ALT-100 mAb in ARDS due to other causes. It is important to note that we previously demonstrated the highly efficacy of ALT-100 mAb in the traumatic blast/VILI-induced lung injury rat model (Bermudez et al., 2022) and in the radiation-induced pneumonitis murine model (Garcia et al., 2022a).

In summary, eNAMPT, a novel cytozyme and DAMP, is a highly druggable ARDS target with biochemical and genomic studies strongly supporting the mechanism of action for the eNAMPT-neutralizing humanized mAb ALT-100 mAb via attenuation of eNAMPT/TLR4 inflammatory cascade activation. We have previously established that eNAMPT mAb delivered with onset of injury is protective in preclinical models using LPS exposure alone (Quijada et al., 2021) and VILI exposure alone (Hong et al., 2008) as well as in LPS/VILI combined challenge models. Our data indicate that delayed delivery of ALT-100 mAb retains high efficacy in attenuating established preclinical sepsis/VILI lung injury, cytokine production, lung fluid imbalance/permeability, and multi-organ failure. These strongly support ALT-100 mAb as a potential strategy to reduce ARDS/VILI mortality. Future research directions will include optimizing dosing and timing for administering the eNAMPT mAb and defining which anti-inflammatory effect is primary or secondary in ARDS i.e., the specific contribution of the mAb to dampening LPS-induced inflammatory amplification loops versus reductions in VILI.

## Data Availability

The raw data supporting the conclusions of this article will be made available by the authors, without undue reservation.
