# Supplementary figures and images for: eNAMPT Neutralization Preserves Lung Fluid Balance and Reduces Acute Renal Injury in Porcine Sepsis/VILI-Induced Inflammatory Lung Injury
Source: Front Physiol. 2022 Jun 22;13:916159. doi: 10.3389/fphys.2022.916159 (PMC9257134; doi:10.3389/fphys.2022.916159)

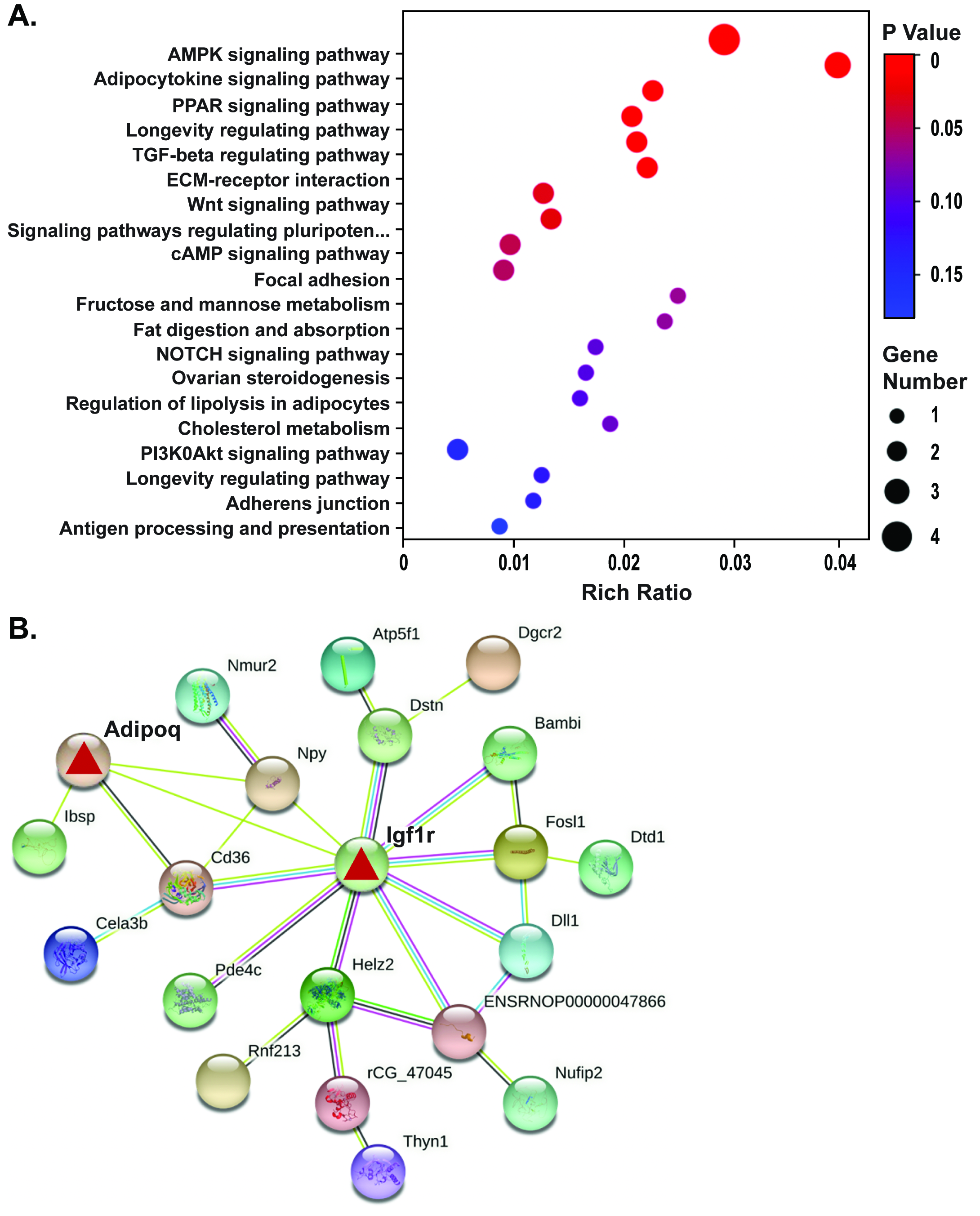

Supplement: Supplementary file 1 [file Image1.TIF]
